# Items

**Published:** 1883-04

**Authors:** 


### Items.

COOK COUNTY HOSPITAL.

We regret to hear that there is no medical clinic on Tuesday.

The surgical clinic Tuesday is conducted with his usual skill by Dr. Fenger.

On Tuesday the medical and surgical clinic is well conducted by Drs. Henrotin and Hutchinson.

The hospital house staff at present consist of Dr. J. A. Fordyce, Chicago Medical College, house physician. His assistants are:

First assistants—Drs. F. S. Johnson, Chicago Medical College; C. E. Currie, Rush Medical College.

Second assistants—Drs. E. P. Davis, Rush Medical College; Elbert Wing, Chicago.

Dr. G. M. Hammon, Rush Medical College, house surgeon. His assistants are:

First assistants—E. R. Bennett, Rush Medical College; J. E. Marshall, Chicago Medical College.

Second assistants—Dr. M. L. Harris, Rush Medical College; W. G. Clarke, Rush Medical College.

The terms of office of the house physician and house surgeon will expire April 1. There are 440 patients in hospital. A complete set of new medical and surgical instruments has just been purchased.

Upon the 8th and 9th of this month was conducted the annual examination of candidates for the position of internes. Applicants to the number of eighteen presented themselves, and submitted papers in the following branches: Anatomy, physiology, chemistry, surgery, ophthalmology and otology, obstetrics, gynæcology, medicine, pathology and materia medica. An hour was given for answering, in writing, five questions from each examiner. The names are presented, in the order of merit, of those who have gained a position upon the house staff:

G. D. Shaver; Clifford B. Wood; Chas. Davison; Thos. E. McDermott; Ernest G. Epler; Wm. H. Weaver; C. M. Coe; Hugh Menzies.

In case any vacancies should occur, the following alternates will have an opportunity to serve. The list is arranged also in the order of merit:

H. M. Hall; Herman E. Burbank; Miss Fannie Dickinson; Miss Isabella White, Frank P. Peck—equal rank.

The advantages presented by this hospital for clinical experience in all departments are so great that those who gain the opportunity to serve as house officers are to be congratulated.

---

THE next annual meeting of the Association of American Medical Editors will be held in the city of Cleveland, Ohio, simultaneously with that of the American Medical Association, on June 5 and 6, 1883. The following will be the order of exercises:

Tuesday, June 5, 7:15 P.M.—Roll called; reading minutes of previous meeting; President's address and discussion thereon; reports of committees; deferred business; new business; election of members; adjournment.

Wednesday, June 6, 7:15 P. M.—Roll called; reading minutes of previous meeting; address by Dr. Henry O. Marcy, of Boston; reading of special papers by Dr. John A. Octerlony, of Louisville, Ky., and Dr. Alexander J. Stone, of St. Paul, Minn.; discussion of addresses and papers; reports of committees; deferred business; new business; election of officers and members; adjournment.

The subject of the address to be delivered by the President, Dr. N. S. Davis, of Chicago, is "The Present Status and Tendencies of the Medical Profession and Medical Journalism." A free discussion upon this important subject is invited, which will be open, not only to members, but to all physicians present. Dr. Marcy's address will be upon the subject of "Journalism devoted to the Protection and Concentration of Medical and Surgical Science in Special Departments."

The Secretary was authorized at the last meeting of the Association, held at St. Paul, Minn., to make the above arrangements for the coming meeting, and also to specially invite all the members of the profession, and friends attending the meeting of the American Medical Association, to be present. The meetings will be held in the interval between the meetings of the sections of the American Medical Association, and the social entertainments of the evening. The sessions will be short, and undoubtedly interesting.

---

COLLEGE OF PHYSICIANS AND SURGEONS.—At the Faculty meeting, March 17, 1883, the following chairs were declared vacant:

Practice of Medicine, occupied by Dr. Carpenter; Surgical Anatomy, occupied by Dr. French; Genito-Urinary Diseases, occupied by Dr. Ielks. At the same meeting the following appointments were made:

Practice of Medicine, Dr. F. L. Wadsworth; Genito-Urinary Diseases, Dr. E. W. Sawyer; Medical Chemistry, Dr. Harrison; Surgical Anatomy, vacant.

Dr. Power resigned the position of Demonstrator of Anatomy.

Dr. Wadsworth has not yet accepted.

THE quarterly meeting of the Illinois State Board of Health will be held in Chicago, April 12, at the Grand Pacific Hotel. At this meeting the annual examination of non-graduates will take place.

DR. S. W. SLEATER, a prominent physician of Charlotte, Michigan, died of congestion of the brain March 18.

THERE are 30 students in attendance at the Woman's Medical College for the spring session.

THERE are 200 students in attendance at the spring session of Rush Medical College.

